# Dose-Dependent Protective Effect of Lithium Chloride on Retinal Ganglion Cells Is Interrelated with an Upregulated Intraretinal BDNF after Optic Nerve Transection in Adult Rats

**DOI:** 10.3390/ijms150813550

**Published:** 2014-08-05

**Authors:** Ming-Mei Wu, Ting-Ting Zhu, Peng Wang, Fang Kuang, Ding-Jun Hao, Si-Wei You, Yao-Yu Li

**Affiliations:** 1 Institute of Neurosciences, The Fourth Military Medical University, Xi’an 710032, China; E-Mails: wumm33@hotmail.com (M.-M.W.); kuangf@fmmu.edu.cn (F.K.); 2 Department of Ophthalmology, General Hospital of Beijing Military Region, Beijing 100700, China; E-Mail: zhutingting85@163.com (T.-T.Z.); 3 Department of Ophthalmology, Taiyuan Aier Hospital, Taiyuan 030012, China; 4 Department of Obstetrics and Gynecology, General Hospital of Beijing Military Region, Beijing, 100700, China; E-Mail: wpfmmu@163.com (P.W.); 5 Department of Spine Surgery, Xi’an Red Cross Hospital, Xi’an 710054, China

**Keywords:** retinal ganglion cell, optic nerve transection, lithium chloride, brain-derived neurotrophic factor, neuroprotection

## Abstract

Neuroprotection of lithium for axotomized retinal ganglion cells (RGCs) is attributed to upregulated intraretinal Bcl-2. As lithium also upregulates brain-derived neurotrophic factor (BDNF) which can rescue axotomized RGCs, it is hypothesized that lithium could protect RGCs through BDNF. This study investigated this hypothesis and a possible relationship between the dose and protection of lithium. All adult experimental rats received daily intraperitoneal injections of lithium chloride (LiCl) at 30, 60 or 85 mg/kg·bw until they were euthanized 2, 7 or 14 days after left intraorbital optic nerve (ON) transection. Our results revealed that RGC densities promoted and declined with increased dose of LiCl and the highest RGC densities were always in the 60 mg/kg·bw LiCl group at both 7 and 14 day points. Similar promotion and decline in the mRNA and protein levels of intraretinal BDNF were also found at the 14 day point, while such BDNF levels increased in the 30 mg/kg·bw LiCl group but peaked in the 60 and 85 mg/kg·bw LiCl groups at the 7 day point. These findings suggested that lithium can delay the death of axotomized RGCs in a dose-dependent manner within a certain period after ON injury and such beneficial effect is interrelated with an upregulated level of intraretinal BDNF.

## 1. Introduction

Lithium has been used as a mood-stabilizer to treat human bipolar disorders for over half a century. In recent years, accumulating evidence demonstrated that lithium is a robust neuroprotective agent for certain central neurons [1,2,3,4,5,6,7,8]. In the visual system, lithium promotes the survival of either retinal ganglion cells (RGCs) *in vitro* and axotomized RGCs *in vivo* after optic nerve (ON) crush in adult mice and rats [9,10,11]. Such protective effects of lithium have been attributed to upregulated expression of Bcl-2 in RGCs [9,10,11]. Brain-derived neurotrophic factor (BDNF) is one of the factors in the neurotrophin family and possesses a marked neuroprotective effect on injured central neurons [12,13,14]. Our previous study showed that olfactory ensheathing cells (OECs) transplanted into the ocular stump of transected ON promoted the number of surviving RGCs in the adult rats and up-regulated intraretinal expression of BDNF was associated with the enhanced RGC survival after OEC transplantation [15]. Our further study demonstrated that BDNF secreted by OECs protected scratch-injured RGCs *in vitro* and BDNF played a major role in such a protection of OECs [16]. Since lithium also upregulates the expression of BDNF in the rat brain [17], we hypothesized that lithium could upregulate the BDNF expression within the retina and protect injured RGCs *in vivo*. Furthermore, different doses of lithium might be able to influence the effects of lithium because it enhanced neurite outgrowth of cultured RGCs in a dose-dependent manner. This study investigated the above hypothesis that lithium could protect axotomized RGCs after rat ON transection through upregulated intraretinal BDNF and explored the possible relationship between the dose and neuroprotective effect of lithium.

## 2. Results and Discussion

### 2.1. Morphology of Surviving RGCs and Glial Cells

**Figure 1:**
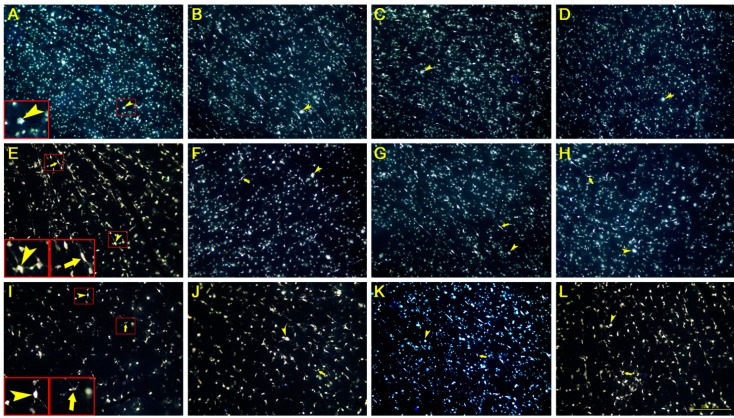
Photomicrographs of surviving retinal ganglion cells (RGCs) retrogradely-labeled with FG in Control 2 days (**A**), 7 days (**E**) and 14 days (**I**) groups; 30 mg/kg·bw LiCl 2 days (**B**), 7 days (**F**) and 14 days (**J**) groups; 60 mg/kg·bw LiCl 2 days (**C**), 7 days (**G**) and 14 days (**K**) groups; and 85 mg/kg·bw LiCl 2 days (**D**), 7 days (**H**) and 14 days (**L**) groups (*n* = 6 for each group). RGCs have large circular cell bodies with clear boundaries (arrow heads). A few glial cells with smaller irregular bodies and short branched processes are also visible in the retina (arrows). Bar = 200 µm.

RGCs of all sizes were labeled with FluoroGold (FG). They had large oval somas and very few processes could be seen with the exception of the proximal part of some primary dendrites. Due to uptake of the dye from dead RGCs, FG-labeled glial cells appeared in the retina 1 week after ON transection. Glial cells had smaller somas and irregular cell borders, and their short and branched processes could be seen clearly. When the RGC number decreased with the survival time, the number of glial cells increased (Figure 1).

### 2.2. Mean Densities of Surviving RGCs

**Figure 2:**
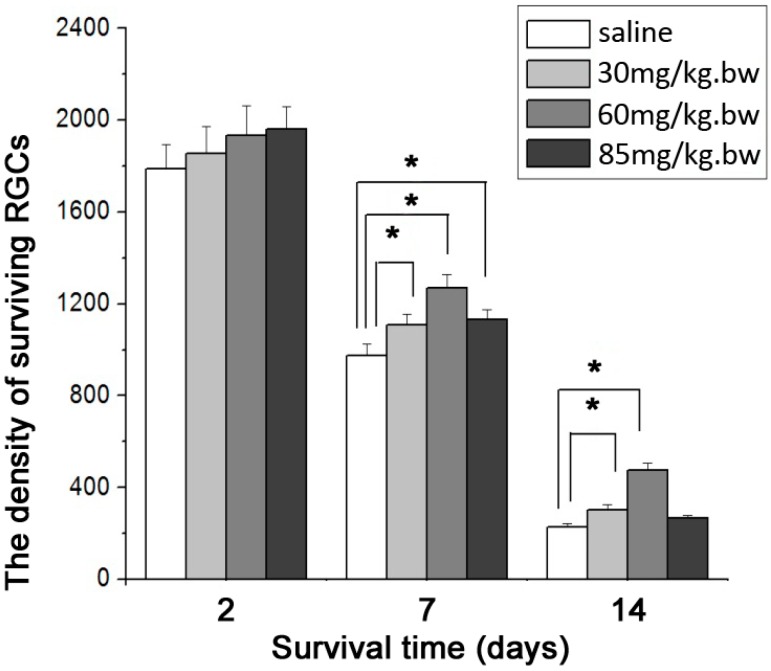
Histogram illustrating the mean densities of surviving RGCs in normal saline (NS)-control, 30, 60 and 85 mg/kg·bw LiCl groups (*n* = 6 for each group) at three different survival time points. The asterisks indicate significantly higher RGC densities (* *p* < 0.01) in LiCl groups compared with those in Control groups at 7 and 14 day time points. Error bars = S.E.M.

The mean densities (RGC number/mm^2^) of FG-labeled surviving RGCs in 30, 60 and 85 mg/kg·bw LiCl experimental groups and normal saline (NS)-treated control group were similar (1853 ± 120, 1932 ± 130, 1961 ± 100 and 1788 ± 106, respectively; *p* > 0.05) 2 days after ON transection. At 7 day point, the RGC densities in 30, 60 and 85 mg/kg·bw LiCl groups (1109 ± 47, 1371 ± 58 and 1131 ± 44) became significantly higher (*p* < 0.01) than that (973 ± 50) in Control group. At 14 day point, the RGC densities in 30 and 60 mg/kg·bw LiCl groups (301 ± 24 and 476 ± 30) remained markedly higher (*p* < 0.01) than that (228 ± 145) in Control group, but the RGC density in 85 mg/kg·bw LiCl group (267 ± 11) was similar (*p* > 0.05) to that in Control group (Figure 2). The highest density was also found in 60 mg/kg·bw LiCl group.

### 2.3. Real-Time Quantitative Polymerase Chain Reaction (RT-PCR) Analysis

**Figure 3:**
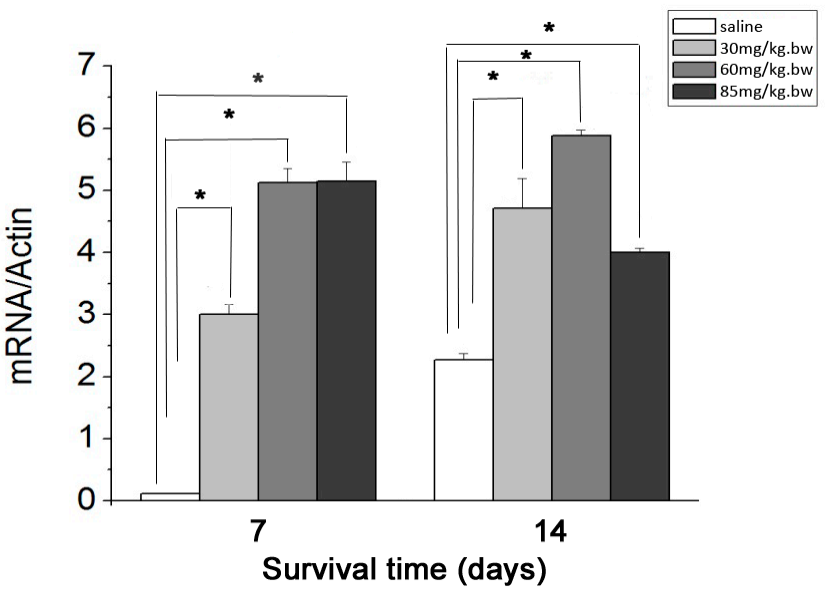
Histogram showing the mRNA levels of brain-derived neurotrophic factor (BDNF) in LiCl and Control groups at 7 and 14 day points. The asterisks indicate significantly higher mRNA levels of BDNF (* *p* < 0.01) in LiCl groups compared with those in Control groups (*n* = 3 for each dosage and time point) at 7 and 14 day points. Error bars = S.E.M.

The mRNA level of BDNF within the retina increased significantly (*p* < 0.01) to the highest level when LiCl dose increased from 30 to 60 mg/kg·bw, and remained similar (*p* > 0.05) afterward as the LiCl dose further increased to 85 mg/kg·bw at 7 day point. The mRNA levels of BDNF at 14 day point were promoted and dropped significantly (*p* < 0.01) in 30, 60 and 85 mg/kg·bw LiCl groups with an increase in the dose of LiCl. The mRNA levels of BDNF in all three LiCl groups were significantly higher (*p* < 0.01) than those in Control groups at both 7 and 14 day points (Figure 3).

### 2.4. Western Blot Assay

**Figure 4:**
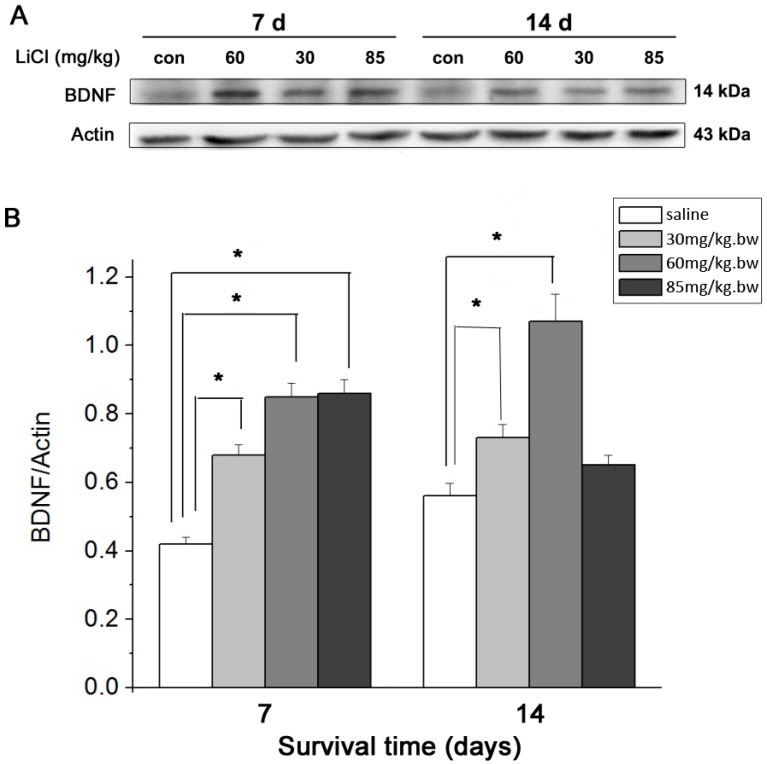
Western Blot assay and densitometric analysis showing the changes of the BDNF protein level at 7 and 14 day time points. (**A**) Representative picture of the immunoblot of BDNF in different LiCl groups and Control groups (*n* = 3 for each dosage and time point) at 7 and 14 day time points; (**B**) Quantitative analysis of Western Blot showing different protein levels of BDNF in LiCl and Control groups (*n* = 3 for each dosage and time point) at 7 and 14 day points. The asterisks indicate higher protein levels of BDNF (* *p* < 0.01) in LiCl groups when compared to those in Control groups at 7 and 14 day points. Error bars = S.E.M.

At the 7 day point, the protein level of BDNF within the retina also increased significantly (*p* < 0.01) to the highest level when the LiCl dose increased from 30 to 60 mg/kg·bw but remained similar (*p* > 0.05) afterward as the LiCl dose further increased to 85 mg/kg·bw. All the BDNF protein levels in these three LiCl groups were significantly higher (*p* < 0.01) than that in the Control group. When the survival time increased to 14 days, the BDNF protein levels were promoted and dropped sharply (*p* < 0.01) in 30, 60 and 85 mg/kg·bw LiCl groups with an increase in the dose of LiCl. When compared to Control group, the protein levels of BDNF were significantly higher (*p* <0.01) in 30 and 60 mg/kg·bw LiCl groups but similar (*p* > 0.05) in 85 mg/kg·bw LiCl group (Figure 4).

**Figure 5:**
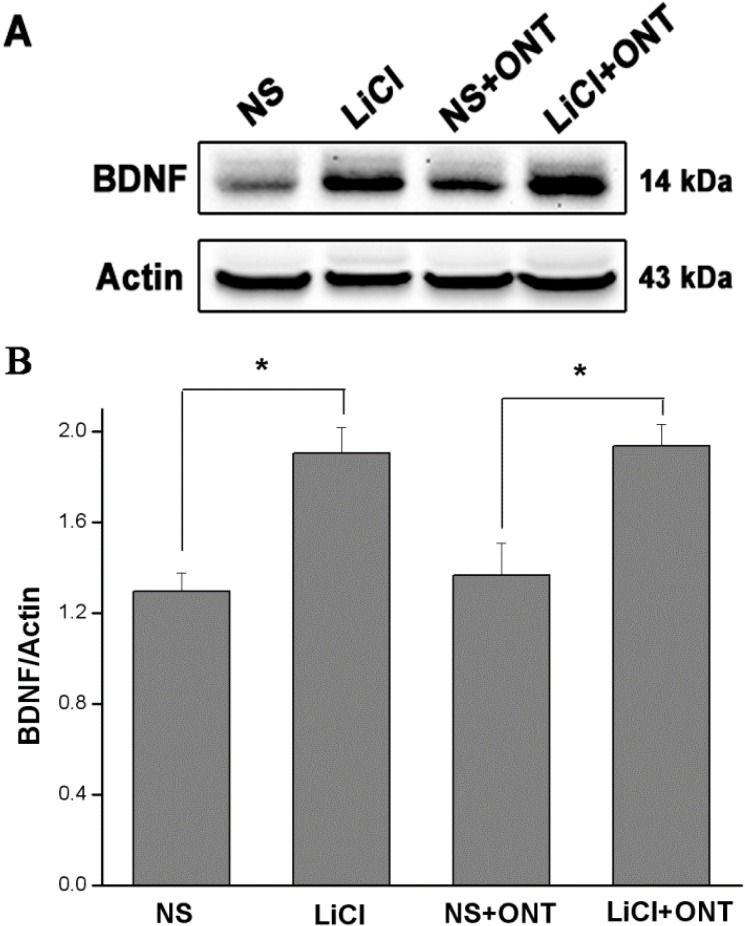
Western Blot assay and densitometric analysis showing the changes of the BDNF protein level of contralateral uninjured eyes and eye with ON transection (ONT) in LiCl (60 mg/kg·bw) and NS-treated groups at the 7 day point. (**A**) Representative picture of the immunoblot of BDNF protein in the LiCl and NS-control groups (*n* = 3 for each treatment and group); (**B**) Quantitative analysis of Western Blot showing different protein levels of BDNF in the LiCl and NS groups. The asterisks indicate higher protein levels of BDNF (* *p* < 0.01) in both the contralateral uninjured eyes and eyes with ON transection in the LiCl groups, when compared to those in the NS-control groups. Error bars = S.E.M.

The protein levels of BDNF were significantly higher (*p* < 0.01) in both the contralateral uninjured eyes and eyes with ON transection in 60 mg/kg·bw LiCl groups, when compared to those in NS-control group at 7 days. However, no significant difference (*p* > 0.05) in the levels of BDNF protein could be found between contralateral uninjured eyes and eyes with ON transection in the 60 mg/kg·bw LiCl groups (Figure 5).

### 2.5. Immunohistochemical Staining of BDNF in the Retina

**Figure 6:**
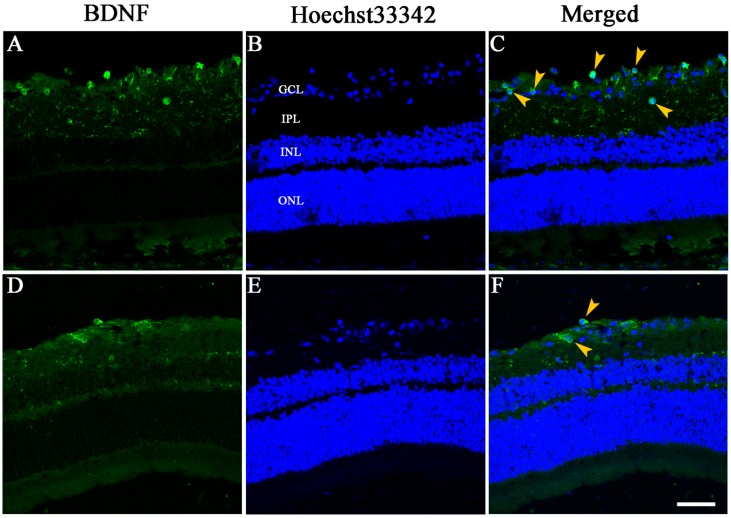
Immunoreactivity of BDNF in radial sections of the rat retina 7 days after ON transection. Stronger BDNF immunohistochemical staining (green) can be found in the GCL and IPL in 60 mg/kg·bw LiCl group (**A**), compared with Control group (**D**, *n* = 6 for each group). The nuclei of cells were stained with Hoechst33342 (blue, **B**,**E**). The arrow heads show BDNF-immunoreactive RGCs in the RGC layer (GCL) in both the LiCl group (**C**) and the Control group (**F**). Scale bar = 50 μm.

Immunohistochemical staining with anti-BDNF antibody showed that BDNF protein expressed in the RGC layer (GCL) and inner plexiform layer (IPL) of the retina in both LiCl (60 mg/kg·bw) and Control groups. Stronger BDNF immunoreactivity was detected in the GCL and IPL in 60 mg/kg·bw LiCl group, compared with the Control group. More BDNF-immunoreactive cells could be found in the GCL in the LiCl group than in the Control group (Figure 6).

### 2.6. Discussion

In the present study, we used the rat model of intraorbital ON transection and followed the previous methods for labeling, counting and calculating the density of surviving RGCs [15]. The number of surviving RGCs begins to reduce about 5 days after intraorbital ON transection [18]; thus, unchanged RGC number or density in the NS-treated control group at day 2 can be used as the control of normal RGC population in this study. When the survival time extended to 7 and 14 days, the population of surviving RGCs usually declined to approximately 50% and 10% of the 2-day RGC population, respectively. In this study, the percentages of surviving RGC densities in Control groups reduced to 52% and 12% at day 7 and 14 day time points, similar to those reported previously [19].

These is no doubt that additional immunohistochemical methods, together with the morphological differentiation of glial cells from FG-labeled RGCs, as well as the quantification of axons in the ON must be more accurate and reliable in differentiating glial cells from RGCs. However, the sole use of injections of FG into bilateral superior colliculi or the ocular stump of transected ON have also been documented in many previous studies [11,20,21] to label the surviving RGCs. Those glial cells and immune cells could hardly be labeled with retrograde FG from the injured ON and glial cells that have phagocytized FG from dead RGCs could easily be recognized in the retina because dying RGCs with changed morphology are still rather different from glial cells. No transneuronal leakage has been reported previously.

Lithium has been widely used as a mood stabilizer for the treatment of human bipolar disorders. During the past years, however, such a common drug was found to have certain *in vitro* and *in vivo* protective effects on various central neurons. For instance, lithium protected cultured cell lines including cerebellar granule cells, cerebral cortical cells, hippocampal neurons and PC12 cells. Lithium provided neuroprotection against a wide variety of insults such as anticonvulsants and potassium deprivation [22,23]. As for the *in vivo* studies, lithium was able to protect cerebral neurons against focal cerebral ischemia, quinolinic acid striatal infusion, excitotoxic lesions of the cholinergic system and kainic acid-induced brain lesions [7,24,25]. In the visual system, lithium rescued RGCs in culture [9] and 30 mg/kg·bw LiCl enhanced the survival of axotomized RGCs 3 weeks after partial ON crush in the adult rats [11]. In the present study, intraperitoneal (i.p.) injections of LiCl at 30, 60 and 85 mg/kg·bw enhanced RGC survival at 7 day time points and such protection remained at the 14 day point when LiCl at 30 and 60 mg/kg·bw was given. Our findings indicated that daily lithium at even higher dosages could still offer a protective effect on axotomized RGCs after complete ON transection in adult rats. However, we could not exclude the possibility that the protective effect of LiCl is transient for a certain period because the longest observing period in this study was only 14 days.

Although the RGC densities in this study continued to be elevated as daily injections of LiCl at 30 and 60 mg/kg·bw were administered, they dropped markedly thereafter when the dose of LiCl further increased to 85 mg/kg·bw at both the 7 and 14 day points. The highest RGC densities were always observed following daily treatments of 60 mg/kg·bw LiCl at these 2 time points. This finding illustrated that the protection of LiCl offered to injured RGCs was presented in a dose-dependent manner and the optimal dose of LiCl for promoting RGC survival was 60 mg/kg·bw. Although the RGC density dropped markedly with LiCl at 85 mg/kg·bw, it was still significantly higher than that in Control group 7 days after ON transection. When the survival time extended to 14 days, however, 85 mg/kg·bw LiCl could no longer promote the survival of injured RGCs. In another study, daily administration of 85 mg/kg·bw LiCl for 1 month also failed to protect injured rubrospinal tract neurons in adult rats [26].

Lithium could increase the level of BDNF expression in cultured rodent cortical neurons [27] as well as in the rat brains [17]. This study further demonstrated that LiCl could increase the expression of BDNF in the rat retina after ON transection and the altered RGC densities with the dose of LiCl at the 7 and 14 day points were highly interrelated with changed intraretinal expression of BDNF. At 7 day point, both mRNA and protein levels of BDNF in the retina became significantly higher than those in Control group when LiCl at 30 mg/kg·bw was used. The intraretinal mRNA and protein levels of BDNF were further raised and thereafter remained unchanged when the dose of LiCl was given at 60 and 85 mg/kg·bw, respectively. Similar elevated mRNA and protein levels of intraretinal BDNF were observed at the 14 day point when the doses of LiCl increased from 30 to 60 mg/kg·bw. However, LiCl at 85 mg/kg·bw resulted in a sharp decline in mRNA and protein levels of BDNF, causing an interrelated drop in the RGC density at the 14 day point.

The previous immunohistochemical and *in situ* hybridization studies demonstrated that BDNF protein expressed in the GCL and IPL of the normal rat retina [28] and BDNF mRNA only expressed in the GCL of the retina after rat ON crush [29]. In order to investigate the types of intraretinal cells that express BDNF in this study, we used anti-BDNF antibody to stain the retinas immunohistochemically in the LiCl (60 mg/kg·bw) and Control groups. Our results revealed that BDNF protein expressed only in the GCL and IPL of the retina 7 days after ON transection in both the LiCl and Control groups, and BDNF immunoreactivity in these two layers looked stronger in the LiCl group than that in the Control group. More BDNF-immunoreactive cells could be detected in the GCL in LiCl group than in the Control group. These findings indicated that LiCl upregulated the expression of BDNF proteins mainly in the GCL of the rat retina after ON transection and those cells that expressed BDNF should most likely be RGCs.

In this study, we found that LiCl increased the number of surviving RGCs and upregulated the level of BDNF in the left retina after ON transection. In order to exclude the possibility of high levels of BDNF being a reflection simply of greater numbers of surviving RGCs, we observed the changes of BDNF level in the uninjured partner eyes of both LiCl-treated and saline-control animals. We found that the BNDF level in the LiCl group was also upregulated significantly in the retina of the contralateral uninjured partner eye which has a stable number of RGCs. Thus, individual BDNF-producing cells should produce a greater amount of BDNF in LiCl treated animals.

Previous studies suggest that the BDNF/TrkB pathway plays an essential role in mediating the neuroprotective effect of lithium. Lithium enhances cell survival by inducing the expression of BDNF, one of the neurotrophins known to regulate neural development, survival and plasticity [30,31]. Lithium selectively promotes the level of exon IV-containing BDNF mRNA and activates the promoter IV of BDNF in neurons [32]. BDNF binds to the TrkB receptor, and thus stimulates certain biological activities in anti-apoptotic pathways [31,33], including phosphatidylinositol 3-kinase/Akt and mitogen-activated protein kinase pathways. Since lithium enhances the neuronal survival and axonal regeneration of RGCs through a Bcl-2-dependent mechanism [9,10,11], the upstream factor BDNF in the present study may protect injured RGCs by eventually activating the Bcl-2 family and the caspases [33] through PI3K/Akt and MEK/ERK pathways, central regulators (GSK-3β) and effector systems (β-catenin, HSF-1, AP-1 and CREB).

## 3. Experimental Section

### 3.1. ON Transection

The left ONs of 138 female Sprague-Dawley rats weighing 200–220 g (Laboratory Animal Center at the Fourth Military Medical University) were exposed via an intraorbital approach under deep sodium pentobarbital anesthesia. After the dural sheath was opened, the ONs were completely transected 1.5 mm from the optic disc using a pair of small iris scissors. Care was taken to maintain the blood supply to the retina intact throughout the operation. A small piece of gelfoam soaked in 5% FG (Fluorochrome, Denver, CO, USA) was applied onto the ocular ON stump to label the surviving RGCs retrogradely.

### 3.2. Treatments and Counting of FG-Labeled RGCs

One day before the left ON transection, 54 experimental animals received daily i.p. injections of LiCl at three different dosages (30, 60 or 85 mg/kg·bw; *n* = 18 for each dosage) until they were killed with an overdose of pentobarbital anesthesia 2, 7 or 14 days after ON transection (*n* = 6 for each dosage and time point). Another 18 control animals received daily i.p. injections of the same volume of NS and survived for 2, 7 or 14 days after ON transection (*n* = 6 for each time point). The left operated eyeballs were enucleated. The retinas were removed, placed in 4% paraformaldehyde in 0.1 M phosphate buffer (PB; pH 7.4) with four radial cuts dividing the retinas into four quadrants, post-fixed in the fixative for 1 h, rinsed in 0.1 M PB, and flat-mounted in 50% glycerin (Sigma-Aldrich, St. Louis, MO, USA ) with the RGC side up on slides. FG-labeled RGCs were counted along the median line of the four quadrants starting from the optic disc to the peripheral border of the retina with an eyepiece grid of 500 × 500 μm^2^ at 500-μm intervals under a BX51 fluorescence microscope (Olympus, Tokyo, Japan) using a 355–425 nm ultraviolet filter. The mean density of surviving RGCs in the whole retina was calculated by averaging the number of all sampled fields in each retina.

### 3.3. RT-PCR Assay

Another 18 experimental animals with daily i.p. injections of LiCl at 30, 60 or 85 mg/kg·bw (*n* = 6 for each dosage) and 6 control animals with the same volume of NS were used to evaluate the intraretinal level of BDNF mRNA. All these experimental and control animals were euthanized at 7 or 14 days after ON transection (*n* = 3 for each dosage and time point). The whole retinas were removed and homogenized in Trizol (Sigma-Aldrich, St. Louis, MO, USA). mRNA was extracted and reverse transcribed with SuperScript II and Oligo (dT) in aPE2400 PCR instrument (PerkinElmer, Irvine, CA, USA). RT-PCR was performed on BDNF, using synthesized cDNA, specific primers and SYBR Green fluorescent marker. The reaction was carried out using Light Cycler 2.0 (Roche Applied Science, Indianapolis, IN, USA) with a 20 µL final volume containing 50 nmol/L of primers, 10 ng of cDNA, nucleotides, Taq DNA polymerase and the SRBR Green I PCR product. The conditions of PCR reaction were initial denaturation phase of 95 °C for 5 min followed by denaturation at 95 °C for 10 s and repeated annealing (40 cycles) at 58 °C for 30 s. The changes in fluorescence of SYBR Green dye in each cycle were monitored by Bio-Rad system software. Gene expression was determined by the Quantification software. The sequences of both the forward and reverse primers used in this study were as follows: β-actin, 5-GGAGATTACTGCCCTGGCTCCTA-3 (forward) and 5-GACTCATCGTACTC CTGCTTGCTG-3 (reverse) and BDNF, 5-GAGCTGAGCGTGTGTGACAG-3 (forward), 5-CGCC AGCCAATTCTCTTTTTGC-3 (reverse).

### 3.4. Western Blot Analysis

Another 18 experimental animals with daily i.p. injections of 30, 60 or 85 mg/kg·bw LiCl (*n* = 6 for each dosage) and 6 control animals with the same volume of NS were used to evaluate the intraretinal level of BDNF protein. All these experimental and control animals were euthanized at 7 or 14 days after ON transection (*n* = 3 for each dosage and time point). Six more animals with left ON transection were killed 7 days after surgery to evaluate the intraretinal level of BDNF protein in the contralateral uninjured right eyes following LiCl or NS treatments (*n* = 3 for each treatment). The whole retina was removed and put in cracking liquid. The protein concentration was determined by the Bradford assay (Bio-Rad, Hercules, CA, USA). A suitable amount of proteins was mixed with 5× protein sample buffer and boiled at 99 °C for 5 min. The boiled proteins were loaded onto a SDS-polyacrylamide gel electrophoresis gel for electrophoresis and transferred to a PVDF membrane (Millipore, Bedford, MA, USA). The membrane was then blocked with 5% non-fat milk at 37 °C for 1 h, washed with Tris-buffered saline containing 0.1% Tween 20 (TBST, Sigma-Aldrich, St. Louis, MO, USA) and processed overnight at 4 °C for immune-staining with a primary antibody against BDNF (1:300; Sigma-Aldrich, St. Louis, MO, USA). After washed with TBST, blots were incubated with the following HRP-conjugated anti-mouse IgG 37 °C for 1 h, and membranes were visualized with the ECL system. The relative densities of the immunoreactive bands were determined and normalized with respect to actin, using a densitometric analysis.

### 3.5. Immunohistochemical Procedures

The BDNF immunohistochemical staining of the retina was used to determine the distribution of intraretinal cells that expressed BDNF after LiCl treatment. Another 6 experimental and 6 control animals with daily i.p. injections of 60 mg/kg·bw LiCl or the same volume of NS were euthanized 7 days after ON transection. Isolated eyes were fixed in 4% fresh paraformaldehyde (pH 7.4) for 1 h, washed thoroughly in 0.1 M PBS and transferred into 30% sucrose solution at 4 °C for 24~48 h until they sank. Cryostat sections of the eyes were cut at 18 µm in thickness, placed on lysine-coated slides and air-dried. Sectioned retinas were treated with a mixture of 0.3% Triton X-100 and 3% bovine serum albumin (fraction V, Sigma-Aldrich, St. Louis, MO, USA) in PBS for 30 min, and then incubated overnight with mouse anti-BDNF (diluted to 1:100, Calbiochem, San Diego, CA, USA) at 4 °C. The sections were rinsed with PBS for 3 times and incubated with the appropriate secondary antibodies (1:1000, anti-mouse IgG, Invitrogen, Grand Island, NY, USA) for 3 h at room temperature. The sections were rinsed with PBS for 3 times again, incubated with Hochest33342 (1:1000, Sigma-Aldrich, St. Louis, MO, USA) for 10 min, rinsed with PBS for 3 times, cover-slipped and examined under a FV 1000 confocal microscope (Olympus, Tokyo, Japan).

### 3.6. Statistical Analyses

Data were analyzed with Bonferroni tests after one-way analysis of variance (ANOVA), using Microcal Origin software (Version 8.0, Originlab Corp., Northampton, MA, USA). Data were presented as mean ± S.E.M. and the significance levels were set to 0.05 for all comparisons.

## 4. Conclusions

Our study suggested LiCl can delay the death of axotomized RGCs in a dose-dependent manner within a certain period after ON transection and this beneficial effect was interrelated with an upregulated intraretinal level of BDNF in adult rats.
